# Psychopathological status, behavior problems, and family adjustment of Kuwaiti children whose fathers were involved in the first gulf war

**DOI:** 10.1186/1753-2000-2-12

**Published:** 2008-05-29

**Authors:** Fawziyah A Al-Turkait, Jude U Ohaeri

**Affiliations:** 1 Department of Psychology, College of Education, Public Authority for Applied Education and Training, Kuwait, P.O. Box 117, Safat, 13002, Kuwait; 2 Department of Psychiatry, Psychological Medicine Hospital, Gamal Abdul Naser Road, P.O. Box 4081, Safat, 13041, Kuwait

## Abstract

**Objectives:**

Following the end of the Gulf War that resulted in the liberation of Kuwait, there are no reports on the impact of veterans' traumatic exposure and posttraumatic stress disorder (PTSD) on their children. We compared the severity of anxiety, depression, deviant behavior and poor family adjustment among the children of a stratified random sample of four groups of Kuwaiti military men, viz: the retired; an active -in-the-army group (AIA) (involved in duties at the rear); an in-battle group (IB) (involved in combat); and a prisoners -of- war (POWs) group. Also, we assessed the association of father's PTSD/combat status and mother's characteristics with child psychosocial outcomes.

**Method:**

Subjects were interviewed at home, 6 years after the war, using: the Child Behavior Index to assess anxiety, depression, and adaptive behavior; Rutter Scale A2 for deviant behavior; and Family Adjustment Device for adjustment at home. Both parents were assessed for PTSD.

**Results:**

The 489 offspring (250 m, 239 f; mean age 13.8 yrs) belonged to 166 father-mother pairs. Children of POWs tended to have higher anxiety, depression, and abnormal behavior scores. Those whose fathers had PTSD had significantly higher depression scores. However, children of fathers with both PTSD and POW status (N = 43) did not have significantly different outcome scores than the other father PTSD/combat status groups. Mother's PTSD, anxiety, depression and social status were significantly associated with all the child outcome variables. Parental age, child's age and child's level of education were significant covariates. Although children with both parents having PTSD had significantly higher anxiety/depression scores, the mother's anxiety was the most frequent and important predictor of child outcome variables. The frequency of abnormal test scores was: 14% for anxiety/depression, and 17% for deviant behavior.

**Conclusion:**

Our findings support the impression that child emotional experiences in vulnerable family situations transcend culture and are associated with the particular behavior of significant adults in the child's life. The primacy of the mother's influence has implications for interventions to improve the psychological functioning of children in such families. Mental health education for these families has the potential to help those in difficulty.

## Background

The first Gulf War (GW) that resulted in the liberation of Kuwait from the Iraqi occupation in early 1991 has given rise to an impressive literature on the issue of posttraumatic stress disorder (PTSD) and co-morbid conditions among the veterans of that war, even in recent times [1,2]. However, the focus has been on PTSD among veterans of the war from the USA and other western nations. In addition, the available literature on the impact of veterans' traumatic exposure and PTSD on their children has been concerned with Vietnam veterans [3-6].

It has been shown that veterans with chronic PTSD suffer both significant intrapersonal and interpersonal difficulties, including problems with family cohesion, self-disclosure, sexual intimacy, and the expression of affection, hostility and aggression [7,8]. These problems are thought to have a negative ripple effect on the wives and children [6,9]. However, psychological characteristics, such as locus of control [10,11] and self-esteem [12-14] can mitigate the expression of the negative impact on families.

In the case of children, they are particularly vulnerable to developing PTSD and other mental disorders (especially anxiety and depression) when exposed to severely traumatic experiences [15-17]. Childhood PTSD is commonly associated with co-morbid mental disorders [18]. The presence of PTSD and violence in veteran trauma survivors has been linked to family dysfunction and symptoms in their children. These include lower self esteem, higher mental disorder rates and symptoms resembling those of the traumatized parent [19]. This has given rise to the suspicion of a transgenerational transmission of effects of war-related trauma [20], which could have a biological basis [21]. Of particular interest in the literature is the impact on child mental status and family adjustment, of veteran's PTSD status and combat exposure, as well as maternal psychosocial distress [5,6,9,15]. These factors were found to interact in such a way as to compromise the child's adjustment. The value of these findings is that they obviate the need to identify children at risk in such potentially provocative home situations and to target them for preventive intervention [15].

A study of psychopathological status, behavior problems and family adjustment among the children of Kuwaiti war veterans is important. First, it will contribute to the scarce literature on how the interaction of GW veterans' PTSD status/combat exposure and their wives' PTSD status impact on their children's psychosocial adjustment. Second, it is an opportunity to examine whether the psychopathological and family adjustment characteristics of these children from a different society that is characterized by being highly conservative (with pronounced male dominance, extended family setting and totally Muslim), transcend cultural barriers by being similar to those of children from the western world. In this regard, it is to be noted that the Kuwaiti society is materially affluent and has an effective national social welfare system. A recent nation-wide epidemiological study showed that Kuwaiti children hail from fairly large, stable and extended family homes (average sibling size of 6.3), with parents predominantly living together (co-habiting is forbidden by law) and fathers gainfully employed, while majority of mothers are housewives[22].

Previous reports on the possible impact of the Gulf War on Kuwaiti children emanated from a general population study [23], as well as studies on personality trait changes, and psychological symptoms among Kuwaiti undergraduate [24] and high school students[25]. In the general population study, the prevalence of PTSD among the children was 10.6% [23]. The study of students revealed significant levels of symptoms of anxiety, depression, somatization, anger, and low self-esteem. However, the findings were not linked to indices of behavior and family adjustment, and the surveys did not include children from military families.

In order to address these issues, we assessed some indices of psychopathology and social adjustment among children of a stratified random sample of Kuwaiti Gulf War veterans, and highlighted the relationship between child and parental psychopathologies. The groups of military men (i.e., fathers) were as follows, in increasing order of war traumatic exposure: a retired group (retired from the army prior to the invasion); an active -in-the-army group (AIA) (i.e., those on duty during the invasion, but involved in duties at the rear only); an in-battle group (IB) (i.e., those involved in actual combat at the fronts); and a prisoners -of war (POWs) group (those imprisoned by the Iraqi forces and released after the liberation). In other words, the POWs were the most exposed to trauma, followed by the IB and AIA, while the retired group was the least exposed.

The specific objectives of the study were as follows:

1. to compare the severity of symptoms of anxiety and depression, as well as behavior abnormalities, poor adaptation, and indices of poor family adjustment among the children of Kuwaiti military men, divided into four groups, as highlighted above. In addition, we highlighted the frequency of probable abnormal test scores for these five conditions.

2. to assess the relationship of fathers' other characteristics (i.e., prevalence of PTSD, co-morbid anxiety/depression, indices of family adjustment, locus of control and self-esteem), on the one hand, with indices of child psychopathology, behavior and family adjustment, on the other hand.

3. to assess the relationship between the mothers' psychopathology (i.e., PTSD, co-morbid anxiety/depression), her social characteristics, such as, number of children, living arrangements (i.e., nuclear family/extended family home), age, employment and educational status, and indices of family adjustment, on the one hand, and the children's psychopathology, behavior and family adjustment, on the other hand.

4. to examine the relationship between fathers'/mothers' PTSD and the children's psychopathology, behavior and family adjustment.

In tandem with the objectives, we hypothesized that fathers' degree of traumatic exposure and PTSD severity would be associated with the severity of psychopathology and poor family adjustment among their children. Specifically, that anxiety and depression scores would be highest among the children of the POWs and IB, as well as the children of men with PTSD (compared with the children of the retired and AIA and the men without PTSD). Similarly, children of mothers with PTSD/anxiety/depression, larger number of offspring, with little or no formal education and living in extended family homes (versus nuclear family homes) would have more severe anxiety/depression scores and poor family adjustment indices [26]. In addition, parents' scores on indices of locus of control, family adjustment and self-esteem would be significantly correlated with their children's scores on indices of psychopathology and family adjustment. Children whose both parents had PTSD would have more severe psychopathological conditions.

## Method

This report concerns only the results of the assessments for the children. The reports on the characteristics of the fathers (i.e., Kuwaiti veterans) [27] and the mothers [28], have been presented in detail elsewhere.

### Selection of subjects and nature of trauma

The Kuwaiti army has only men in its service. The method for selecting the military families has been described in detail elsewhere [27,28]. It should be noted that, although the military groups were chosen to represent degrees of exposure to the trauma of war, all Kuwaitis had potential to be exposed to psycho-trauma during the occupation [2,28].

### Instruments for assessing the parents

Among the instruments used to interview the parents were the following: (i) the Clinician Administered PTSD Scale (CAPS) (for the fathers) – for DSM-IV diagnosis of PTSD [29]; (ii) the Hopkins Symptom Checklist -25, to screen for anxiety and depression (HSCL -25) [30]; (iii) internal-external locus of control (I-E LOC) [31]; (iv) the 10-item Self-esteem Scale (SES) [32]; (v) the McMaster Family Assessment Device (FAD) [33]; and (vi) the PTSD Checklist (PCL) (for the mothers) – for ascertaining probable DSM-IV PTSD [34,35].

Details about these questionnaires have been presented elsewhere [27,28].

### Instruments for assessing the children

The children were assessed with three instruments (see below for details), viz:

The McMaster Family Assessment Device (FAD) [33] was administered in face-to-face interview, only to children who were over 12 years of age (N = 281). This is in line with standard guidelines for using the questionnaire. Similarly, the Child Behavior Inventory (CBI) and Rutter Scale A-2-parent's version were used to assess child anxiety/depressive symptoms and behavioral problems, respectively. For the CBI, the questionnaire was completed by interviewing mothers of children below 10 years of age, while children aged 10–16 years were interviewed face-to-face. Also for the Rutter Scale, only children aged 6 – 16 years were assessed, as recommended (i.e., N = 355 for CBI and Rutter Scale interviews).

### The Family Adjustment Device (FAD) [33]

This is a screening instrument to identify problem areas in the most simple and efficient manner. It is based on the assumption that family functioning is much more related to transactional and systematic properties of the family system than to intra-psychic characteristics of individual family members. It was designed to avoid genuine differences in view, where the family may not be perceived in the same way by observers with different points of view. The 53 items are statements a person could make about his/her family. Each family member rates his/her agreement with how well an item describes the family by selecting among the four response options: strongly agree, agree, disagree and strongly disagree. Higher scores indicate unhealthy family adjustment. The FAD is made up of seven subscales which measure the individual's perception of how well the family is adjusted in the following domains: Problem Solving, Communication, Family Roles, Affective Responsiveness, Affective Involvement, Behavior Control and General Functioning. The subscale labels are indicative of their underlying constructs. For example, problem solving refers to the family's ability to resolve issues which threaten their integrity and functional capacity. Communication refers to the exchange of information among members. The dimension, Roles, focuses on whether the family has established patterns of behavior for handling a set of family functions, including provision of resources, nurturance and support [33]. In view of the absence of standard cut-off scores, it is recommended that abnormal test scores should be judged by the group mean plus one standard deviation.

### Child Behavior Inventory (CBI) [36]

The scale was designed to assess children's anxiety, depression and behavioral symptomatology following experience of traumatic events of war. The English version has 43 questions. The measure has been translated into Arabic, and has been adapted for use in Lebanon and Kuwait [23]. The Kuwaiti version has 42 items. Before its use in Kuwait, the CBI was pilot-tested to assess the meaning and relevance of the questionnaire items for Kuwaiti children. The items are grouped into five domains: aggression, depression, anxiety, prosocial and planful behavior. Each domain is represented by a set of questions that inquire about the child's behavior six months prior to the assessment. The five domains are also grouped into two main headings: a) mental health symptoms of aggression, depression, and anxiety; b) adaptational outcomes of prosocial and planful behavior.

### Mental health symptoms

(i) Aggression (9 items: a maximum score of 27): e.g., gets angry easily, verbally aggressive, physically aggressive towards others, destroys his/her or other peoples things, etc.

(ii) Depression (9 items: a maximum score of 27): e.g., appears sad or unhappy, distances him/her self from love and care, etc.

(iii) Anxiety (6 items: a maximum score of 18): e.g., jumpy, indicates that he/she is frightened that something bad will happen to him/her, reacts with fear to things or situations that do not usually scare other children, etc.

### Adaptational outcomes

(i) Prosocial behavior (9 items: a maximum score of 27): e.g., helpful towards other children, helpful towards adults, shows concern or cares for others, etc.

(ii) Planful behavior (9 items: a maximum score of 27): e.g., takes the lead in initiating activities, plans and thinks ahead, skillful in solving problems, etc. Each question is scored on a four-alternative, forced-choice format, ranging from 0 = never, through, 1 = rarely and 2 = sometimes, to 3 = always.

Higher scores for the mental health items indicate pathology, while for the adaptational outcome items, higher scores indicate positive adaptation.

### Rutter A-2 Scale – Parents' version [37]

This scale, which is a slightly modified version of the original form A, consists of 31 statements concerning the child's behavior. The mother rated the extent to which the statement applied to the child. The scale is divided into 3 parts:

(i) Health problems (8 items): e.g., headache, stomach-ache, wets bed, temper tantrums, truants from school, etc. The subscale score is 0–16.

(ii) Habits (5 items): e.g., stammers/stutters, steals things, eating problems, etc. The subscale score is 0–10.

(iii) Statements on behavior (18 items): e.g., restless, destroys own or others' belongings, fights with others, has twitches, mannerisms or tics, sucks thumb or finger, disobedient, tells lies, bullies other children, etc. The total score is 0–36.

The most prominent behavioral problems that can be extracted from these 18 statements are:

(i) Neurotic: the following are scored for a neurotic subscale: tears on arrival at school, sleep problems, worried and fearful.

(ii) Antisocial: the following are scored for the antisocial subscale: steals things, destroys own or others' belongings, disobedient, tells lies, bullies other children.

Each item is scored on a scale of 0, 1 or 2. The subscale scores are computed by adding the ratings for each item. Higher scores indicate pathology.

The Arabic version of the above questionnaires (produced by back-translation), has been used by previous workers in the Kuwaiti and neighboring Arab populations, and the contents were found to be relevant to the respective constructs and easily understood by Arabs [23,38,39]. We note that these instruments are not meant to be diagnostic of the various underlying constructs, but give indication of severity of probable problems in the respective domains.

### Reliability coefficients

The internal consistency of the questionnaires was assessed by Cronbach's alpha and Guttmann's split- half coefficient, using the responses of all the subjects. The alpha coefficients were above the recommended 0.7. For the CBI, Rutter Scale and FAD, the alpha values were, respectively, 0.92, 0.85 and 0.76.

### Construct validity – Factor analysis for the CBI and Rutter's Scale

In view of the wide cultural difference between Kuwait and the western world where the questionnaires were originally articulated, it was necessary to examine whether the responses of our subjects would yield similar domains as in the original questionnaires. We used factor analysis with principal component analysis and varimax rotation for factors with eigen values above one. This analysis was not done for the FAD because our sample size (N = 281 for subjects aged > 12 years) was not considered adequate for this analysis, since the FAD has 53 items.

For the CBI and Rutter Scale, the original constructs of the questionnaires were adequately replicated, with the items loading highly (> 0.45) on their respective factors (data available on request from the authors).

### Procedure

As a result of the national security situation at that time (the old regime in Iraq continually threatened the sovereignty of Kuwait), and the difficulty of obtaining permission for the study from the military authorities, coupled with the conservative nature of the society, and the problem of contacting the sampled subjects, it was six years after the GW that the study could commence. Ethical approval was obtained from the Public Authority for Applied Education and Training, Kuwait, and the Ministry of Defense, Kuwait. All responding veterans gave written informed consent for their wives and children to be interviewed. Accordingly, the rest of the family agreed to be interviewed. In the Kuwaiti culture, the father's consent for such a non-invasive exercise is a sufficient reason for the remainder of the family to participate.

The interviews were conducted by eight Arab female psychology graduates, who were employed in the mental health service as psychologists/social workers, and had previous experience in interviewing people for social science/mental health research. At the preliminary stage of the study, the principal investigator trained the research assistants for one week by lectures and practical demonstrations in the technique of interview. They took turns to read and rate the responses of patients at the special PTSD clinic (Al-Riggae Center), and were thereby able to harmonize their ratings. The formal study began when the investigator was satisfied that the research assistants had achieved satisfactory inter-rater reliability of ratings. Unfortunately, no formal inter-rater reliability tests were done. However, at monthly intervals, the research team met to jointly rate subjects and ensure that interviews were being done correctly. After the period of training, the research team conducted a pilot study with the families of ten soldiers (not part of the main study), who were receiving treatment for PTSD at the Al-Reggae Center, at their homes. It was found that, although the interview lasted an average of two hours for each family, the relaxed atmosphere at home and the manner in which the subjects had been approached, made the exercise acceptable to the subjects. Respondents were not compensated for the interviews, as the cultural norm does not support material inducements for such activities. Different research assistants interviewed the husband, wife and children, and each respondent was interviewed privately, in order to avoid bias in ratings.

Each prospective respondent soldier was firstly contacted by telephone, and according to his choice, the family was interviewed either at his home in the evenings, or at the Al-Riggae Center. This report concerns the results of interviews with the children only.

### Data analysis

Data were analyzed by SPSS version 11. The total scores for the following child outcome variables were computed by summing up the scores of the corresponding subscales of the questionnaires: Child Behavior Inventory (CBI) anxiety, CBI depression, CBI aggression, CBI prosocial behavior, CBI planful behavior; Rutter Scale (RS) health problems, RS habits, RS statements of behavior, RS neurotic, RS antisocial; Family Adjustment Device (FAD) Roles, FAD Response, FAD communication, FAD involvement, and FAD general.

For the first objective, we used one-way ANOVA to compare the scores on child outcome variables across father's combat exposure levels. Effect sizes were also calculated. In view of the fact that the three instruments for assessing the children have no standard cut-off scores for Kuwait, and the data were fairly normally distributed, probable abnormal test scores were judged by the following: scores greater than the group mean plus 1 SD for CBI depression/anxiety/aggression/Rutter/FAD; and less than the group mean plus 1 SD for CBI prosocial/planful.

For the second and third objectives, we used t-test and effect size to compare scores in child outcome variables, between those whose parents had PTSD and those whose parents did not have PTSD. Similarly, we assessed differences in child outcome variables for the different categories of parental socio-demographic characteristics (e.g., employment status, nuclear/extended family home). Furthermore, we used Pearson's correlation to assess the relationship between child outcome variables and parental characteristics, such as age, and scores on self-esteem and locus of control. In view of the many significant relationships in the above univariate tests, we used multiple regression analyses to determine the parental characteristics that could predict child outcome variables. For this analysis, each child outcome variable (e.g., CBI anxiety, CBI depression score) was used as the dependent variable, while parental continuous variables (e.g., age, PTSD severity score, anxiety/depression scores) were used as independent variables.

For the fourth objective, we grouped the children, first according to categories of father versus mother combinations of PTSD status (e.g., father has PTSD and mother has PTSD; both parents do not have PTSD, etc). Second, we grouped the children according to categories of father's PTSD status versus combat exposure combinations (e.g., father is retired and had no PTSD; father was POW and had PTSD, etc). We used two-way ANOVA (general linear model) to assess the interactions of father – mother PTSD and father's PTSD – father's combat exposure on child outcome variables. In the post-hoc tests that followed the two-way ANOVA operations, we used one-way ANOVA to assess group differences in child outcome variables. In view of the differences in father's age, as well child's age and level of education (by level of trauma exposure groups), the association of parental characteristics with child outcome variables was also assessed by analysis of covariance (using parental age, child's age and child's education as covariates).

Where multiple tests were done, the level of significance was set at P < 0.01 (Bonferroni correction); otherwise, the P level was 0.05. All tests were two-tailed.

## Results

### Socio-demographic characteristics

Of the 200 veterans assessed, 187 were married and 166 wives had children.

We defined a child as one who was still living at home, never married and never earned a salary. Thus, the 489 (51.1% m, 48.9% f) children who fulfilled these criteria belonged to 166 military father and 166 mother pairs. On the whole, however, the mothers had an average of 4.6 (SD 2.2) children. The mean age of the children was 13.6 (SD 5.4) years (range 6–33). Majority (252 or 51.5%) were aged 11–20 years, 174(35.6%) were aged 6–10 years, 51 (10.4%) were aged 21–25 years, 10 (2.0%) were aged 26–30 years, while only 2 (0.4%) were aged over 30 years. All the children had some level of education: 139 (28.5%) were in primary school, 274(56.1%) were in high school, and 75 (15.4%) were studying for diploma/university degrees. Mean age did not differ by gender (M = 13.5, F = 13.7, P = 0.7), and level of education was similar by gender (P = 0.3). However, the children of the retired men were significantly older (F = 34.6, df = 3/485, P < 0.001) and had higher educational attainments (X^2 ^= 130, df = 4, P < 0.001) than the other groups.

According to fathers' level of combat exposure, the 489 children were sorted into the following categories: children of the retired, 183 (37.4%); children of the active-in-army (A-I-A), 102 (20.9%); children of the in-battle (IB), 103 (21.1%); and children of the POWs, 101 (20.7%). However, following standard recommendations for using the instruments, the CBI and Rutter Scale were applied to only the 355 children aged 6–16 years, while the FAD was applied to only the 281 children aged above 12 years.

### Frequency of probable abnormal test scores and co-morbidity for the subscales of the three child outcome instruments (Table 1)

**Table 1:** Frequency of abnormal test scores for the CBI (N = 355), Rutter Scale (N = 355) and FAD (N = 281)*

Rating scale's subscale label	No. of children with abnormal test scores	%
CBI depression	51	14.4
CBI anxiety	53	14.9
CBI aggression	57	16.1
CBI prosocial behavior	59	16.6
CBI planful behavior	70	19.7
Rutter neurotic	60	16.9
Rutter antisocial	61	17.1
FAD problem solving	27	9.6
FAD communication	50	17.8
FAD roles	65	23.1
FAD responsiveness	31	11.0
FAD involvement	47	16.7

* Abnormal test scores judged by: scores > group mean + 1 SD for CBI depression/anxiety/aggression/Rutter/FAD; and < group mean + 1 SD for CBI prosocial/planful)

Using the group mean (+/- 1 SD) as cut-off scores, we found that 14.4% and 14.9% had probable clinical severity of depression and anxiety, respectively. In addition, 17.1% evidenced a tendency for antisocial behavior, 9.6% – 23.1% indicated significant problems in family adjustment, and 16.6%–19.7% probably had problems in adaptational behavior. Furthermore, anxiety/depression co-morbidity was a common feature. Hence, 27(52.2%) of those with probable clinical depression also had clinical anxiety, and 27(50.9%) of those with probable clinical anxiety also had clinical depression (X^2 ^= 67.8, df = 1, P < 0.0001, in each case). Clinical depression was highly significantly associated with child's aggressive behavior (X^2 ^= 37.3, df = 1, P < 0.0001), deficient prosocial behavior (X^2 ^= 9.4, df = 1, P < 0.002), and deficient planful behavior (X^2 ^= 5.1, df = 1, P < 0.002). Similarly, clinical anxiety was significantly associated with child's aggressive behavior (X^2 ^= 34.6, df = 1, P < 0.001) and deficient prosocial behavior (X^2 ^= 6.1, df = 1, P < 0.01). However, child clinical anxiety and depression were not significantly associated with the probability of having significant family adjustment problems (P > 0.05).

### Association of father's combat exposure and PTSD status with child's outcome variables (Table 2)

**Table 2:** Groups with significant differences in psychopathological, behavioral and family adjustment scores, by father's combat exposure

Variables	Military status or combat exposure of fathers: Mean (SD), DF = 3/351							
	Retired (1) (N = 93)	Active-in Army (2) (N = 86)	In- battle (3) (N = 85)	POWs (4) (N = 91)	F	P	Significantly different groups	Effect size (95% C.I.)

Rutter statements on behavior	5.7 (5.4)	5.4 (5.0)	5.6 (4.7)	7.5 (5.5)	3.5	0.025	4 > 2	0.40 (0.1–0.69)
CBI – Depression	5.7 (4.9)	6.8 (5.1)	5.0 (3.3)	7.4 (5.2)	4.9	0.003	4 > 1; 4 > 3	0.34 (0.04–0.63);0.54 (0.24–0.85)
CBI – prosocial	16.3 (5.7)	15.1 (7.2)	14.5 (4.5)	17.3 (5.4)	4.2	0.006	4 > 3	0.56 (0.26–0.86)
FAD subscales	(N = 133)	(N = 45)	(N = 51)	(N = 52)			DF = 3/277	
FAD problem	1.9 (0.5)	2.0 (0.4)	2.2 (0.3)	1.9 (0.3)	5.1	0.002	3 > 1	0.66 (0.33–0.99)
FAD communication	2.2 (0.4)	2.3 (0.4)	2.4 (0.3)	2.3 (0.3)	5.7	0.001	3 > 1	0.53 (0.20–0.86)
FAD Roles	2.4 (0.4)	2.5 (0.2)	2.4 (0.4)	2.4 (0.3)	4.7	0.003	2 > 1	0.28 (-0.06–0.62)

Children of POW veterans consistently tended to have higher anxiety, depression and abnormal behavior scores, while having higher adaptational scores (CBI adaptation). These trends reached significance for the following: (i) for depression: the POW group scored significantly higher than the retired and IB (P < 0.003); (ii) for Rutter Statements on behavior, the POW group scored significantly higher than the AIA (P < 0.03); and (iii) for prosocial behavior, the POW group had higher scores than the IB group (P < 0.006). In the case of family adjustment, the children of retired veterans tended to have more positive adjustment scores. This tendency reached significance for family problem solving and communication (versus the IB group) (P < 0.001), and for FAD Roles (versus AIA) (P < 0.003).

With regard to father's PTSD status, the only significant difference was for child's CBI depression. Those whose fathers had PTSD (N = 105) scored significantly higher (7.3, SD 5.1), than those whose fathers did not have PTSD (N = 250) (5.8, SD 4.6; t = 2.6, df = 353, P = 0.01) [Effect size & 95% C.I. = 0.32 (0.09–0.54)].

### Interaction of father's PTSD status and combat exposure (Tables 3 &4)

**Table 3:** Prevalence of combined groups of father's PTSD status and military status (N = 489)

No PTSD & Retired (1)	154 (31.5%)
No PTSD & Active-in-Army (2)	64 (13.1%)
No PTSD & In-battle (3)	67 (13.7%)
No PTSD & POW (4)	59 (12.1%)
PTSD & Retired (5)	35 (7.2%)
PTSD & active-in-army (6)	38 (7.8%)
PTSD & in-battle (7)	29 (5.9%)
PTSD & POW (8)	43 (8.8%)

**Table 4:** Interaction of father's PTSD status and military status on child's psychopathological, behavioral and family adjustment variables

Variables	Two-way ANCOVA*: Interaction statistics		Post – hoc tests		
	F	P	F	P	Groups in Table 3 with significant difference (& level of significance)

Rutter statements on behavior	1.4	0.25	2.7	0.01	4 > 2 (0.008); 4 > 1 (0.03)
Rutter discriminant	3.8	0.01	2.9	0.005	4 > 3(0.04); 4 > 2(0.025); 4 > 1(0.01); 5 > 1(0.04)
CBI depression	4.7	0.004	5.4	0.000	4 > 1(0.002); 4 > 2(0.04); 4 > 3(0.001); 5 > 3(0.02); 6 > 3 (0.003)
CBI anxiety	2.2	0.09	2.7	0.01	4 > 1(0.002); 4 > 2(0.03)
CBI prosocial	4.0	0.009	3.3	0.02	4 > 1(0.04); 4 > 2(0.003)
FAD problem solving	0.1	0.94	2.4	0.02	3 > 1(0.007)
FAD communication	1.4	0.24	3.6	0.001	3 > 1(0.005); 7 > 1(0.01)
FAD Roles	3.3	0.02	4.0	0.000	3 > 5(0.006); 2 > 5(0.003)

* Adjusted for age of fathers and children.

Although there was significant interaction between father's PTSD status and combat exposure in two- way ANOVA, the post hoc tests showed that, of the 43(8.7%) children whose fathers were both POWs and had PTSD, there was no significant tendency for them to score higher than the children in other groups on indices of child psychopathology, behavior and family adjustment (Tables 3 &4). But the POW status (without PTSD) was commonly associated with higher scores in depression, anxiety, Rutter Statements on behavior, Rutter discrimination, and prosocial behavior, compared with the other groups. However, there was a consistent tendency for the children whose fathers were both retired and had no PTSD, to score least on psychopathological and abnormal behavior indices, while having better family adjustment indices. The in-battle group was significantly associated with abnormal family adjustment indices, compared with the retired (P < 0.01). In ANCOVA, with father's age, child's age and child's education as covariates, the above differences in Rutter Statements on behavior, CBI depression and anxiety were no longer significant (P > 0.05). But the findings for prosocial behavior (POW > AIA; P < 0.04), as well as poor family adjustment indices for the in-battle group, remained significant (P < 0.01).

### Relationship with mother's PTSD status (Table 5)

**Table 5:** Groups with significant differences by mother's PTSD status

Variables	Mother has probably no PTSD (N = 259)	Mother has probable PTSD (N = 92)	T	P	DF	Effect size (95% C.I)
CBI – depression	5.8 (4.6)	7.4 (9.4)	2.9	0.004	349	0.26 (0.02–0.50)
CBI aggression	5.3 (5.0)	7.6 (5.2)	3.8	0.000	349	0.46 (0.21–0.69)
CBI anxiety	5.9 (3.7)	8.0 (3.6)	4.9	0.000	349	0.57 (0.33–0.81)
CBI planful	13.3 (6.1)	11.3 (5.2)	2.9	0.004	349	0.34 (0.10–0.58)
Rutter statements on behavior	5.6 (4.9)	7.1 (5.8)	2.3	0.02	349	0.29 (0.05–0.53)
Neurotic	1.1 (1.3)	1.8 (1.5)	3.8	0.000	349	0.52 (0.27–0.76)
FAD subscales	(N = 209)	(N = 69)				
Problem solving	1.9 (0.4)	2.1 (0.3)	2.3	0.02	276	0.53 (0.39–0.94)
Communication	2.2 (0.3)	2.4 (0.3)	3.2	0.002	276	0.67 (0.42–0.91)
General	2.3 (0.3)	2.4 (0.3)	2.2	0.029	276	0.33 (0.06–0.61)

Mother's PTSD status had significant association with all the child outcome variables. Hence children of mothers with PTSD had significantly higher scores for CBI anxiety, depression, and aggression; lower scores for CBI planful behavior (i.e., were less motivated); higher scores for the Rutter subscales (i.e., abnormal behavior) (P < 0.01); and poorer family adjustment scores (P < 0.02).

### Interaction of father's and mother's PTSD (Table 6)

**Table 6:** Interaction of father's and mother's PTSD: groups with significant differences.

							Df = 3/351 for CBI		
Child outcome variables	NF* & NM(1) (N = 192) Mean(SD)	YF & NM(2) (N = 71) Mean(SD)	NF & YM(3) (N = 58) Mean(SD)	YF & YM(4) (N = 34) Mean(SD)	Two-way ANCOVA**: Interaction statistics		Post – hoc tests		
					F	P	F	P	Significantly different groups

CBI – Neurotic	1.1(1.3)	1.3(1.4)	1.8(1.2)	1.7(1.9)	1.9	0.16	5.2	0.002	3 > 1: 4 > 1
CBI – depression	5.4(4.5)	7.0(4.9)	7.3(4.6)	7.7(5.7)	0.2	0.68	4.8	0.003	3 > 1: 4 > 1
CBI – anxiety	5.9(3.6)	6.1(3.7)	8.3(3.3)	7.7(4.0)	0.3	0.56	8.0	0.000	3 > 1: 4 > 1
CBI – aggression	5.2(4.9)	5.8(5.5)	7.4(4.7)	7.9(6.0)	0.03	0.86	4.7	0.003	3 > 1: 4 > 1
CBI – planful	13.1(6.4)	14.0(5.0)	11.3(5.4)	11.3(4.9)	0.08	0.78	3.2	0.02	2 > 3
FAD subscales:	(N = 156)	N = 56)	(N = 43)	(N = 26)					Df = 3/227
Problem solving	1.9(0.4)	2.1(0.4)	2.2(0.3)	2.0(0.3)	3.5	0.06	3.7	0.01	3 > 1
Communications	2.2(0.4)	2.3(0.4)	2.5(0.3)	2.2(0.1)	7.1	0.009	8.5	0.000	3 > 1; 2 > 1
Involvement	2.4(0.4)	2.3(0.4)	2.5(0.4)	2.7(0.3)	8.2	0.005	6.7	0.000	4 > 1; 1 > 2
General	2.3(0.3)	2.3(0.3)	2.4(0.3)	2.5(0.2)	2.2	0.14	2.7	0.046	4 > 1

Notes: *NF & NM = Father has no PTSD and mother has no PTSD

YF & NM = Father has PTSD and mother has no PTSD

NH & YM = Father has no PTSD and mother has PTSD

YF & YM = Father has PTSD and mother has PTSD

** Adjusted for age of child and father

Although there was no significant interaction between parents' PTSD status, the post hoc tests showed that, children whose mothers had PTSD or both parents had PTSD, consistently tended to have higher psychopathological, abnormal behavior and poorer family adjustment scores, in comparison with those whom both parents did not have PTSD. This tendency reached significance for CBI depression (P < 0.003), anxiety (P < 0.001), aggression (P < 0.003), FAD communications, and involvement (P < 0.001). However, when the data were subjected to ANCOVA, with the parent's age, child's age and child's level of education as covariates, the differences were no longer significant for the following: CBI depression, CBI aggression, CBI planful, Rutter neurotic, FAD roles and FAD general. The findings for CBI anxiety (P < 0.03), FAD problem (P < 0.04) and FAD communication (P < 0.003) remained significant.

### Correlation of child outcome variables with parent's psychopathological and FAD scores

Using Pearson's correlations, we found that the relationships between child and parental variables that reached significance level of P < 0.001, were mostly with regard to the mother. Hence, child psychopathological, behavioral and family adjustment scores were more commonly highly significantly correlated with mother's PTSD, anxiety and depression scores, compared with father's scores (Pearson'r for mother's anxiety/depression versus child's scores: mostly > 0.30, P < 0.0001). This is in line with Tables 5 and 6.

### Multiple regression analyses (Table 7)

**Table 7:** Predictors of child's psychopathological, behavioral and family adjustment variables: multiple regression analyses

Dependent variables	Predictors (Independent variables)	Variance (%)	Total variance	B	T	P
CBI – depression N = 355 for all CBI subscales	Mother's anxiety	9.1	15.0	0.29	5.6	0.000
	Child's age	2.7		0.18	3.4	0.001
	Father's military status	1.8		0.13	2.4	0.016
	Father locus of control after war	1.4		0.12	2.3	0.023
CBI – anxiety	Mother's anxiety	16.8	22.0	0.42	8.4	0.000
	Father's anxiety	1.4		-0.34	-4.2	0.000
	Father's depression	1.7		0.21	2.6	0.009
CBI adaptation	Father's PTSD severity	10.5	18.5	0.33	6.4	0.000
	Father's anxiety	1.9		-0.20	-2.5	0.012
Rutter total score N = 355 for all Rutter Subscales	Mother's anxiety	10.5	18.5	0.33	6.4	0.000
	Father's anxiety	1.8		-0.38	-5.0	0.000
	Father's PTSD severity	3.9		0.29	4.0	0.000
	Father's LOC pre-war	1.2		0.12	2.6	0.025
	Child's education	1.1		0.11	2.1	0.36
Neurotic	Mother's anxiety	13.5	16.5	0.37	7.4	0.000
	Education of child	2.0		0.15	2.9	0.003
Antisocial	Mother's anxiety	6.2	9.2	0.25	4.6	0.000
	Father's anxiety	1.2		-0.25	-3.3	0.000
	Father's PTSD severity	1.9		0.20	2.6	0.01
FAD communication: N = 281 for all FAD Subscales	Mother's depression	2.2	5.2	0.38	2.9	0.004
	Father's LOC pre war	1.5		0.12	2.0	0.045
	Mother's anxiety	1.5		-0.26	-1.9	0.048
FAS Roles	Father's military status	6.4	6.4	0.25	4.2	0.000
FAD response	Father's PTSD severity	2.7	2.7	-0.17	-2.7	0.008
FAD involvement	Mother's anxiety	3.6	9.2	0.21	3.5	0.001
	Father's depression	2.3		-0.38	-3.9	0.000
	Father's anxiety	3.2		0.29	3.0	0.003
FAD general	Mother's depression	4.2	4.2	0.20	3.4	0.001

The above findings (i.e., Tables 5 &6) were supported by the results of the multiple regression analyses. Table 7 shows that the commonest and most important predictors of child outcome variables were the mother's anxiety and depression. Hence, of the 11 child psychosocial outcome variables, mother's anxiety accounted for the majority of variance in six, while mother's depression accounted for the majority of the variance in two. Father's PTSD/combat exposure accounted for the majority of the variance only in the case of adaptive behavior and the roles/response subscales of the FAD.

## Discussion

### Limitations and strengths of the study

The major limitations of the study are that we did not use diagnostic instruments, and we did not specifically assess the impact of social supports. Furthermore, we did not assess the possible influence of child cognitive capacity and personality, which are thought to be important determinants of psychological vulnerability after trauma [40]. However, our instruments are time-tested, of wide international use, and have been found to be valid and reliable in previous studies in Kuwait and neighboring states [23,36,38,39]. In addition, the scales in the instruments showed very good internal consistency and validity. The acceptability of the questionnaires and the interview process is shown by the low refusal rate (4% of soldiers contacted), and the fact that all those who consented to be interviewed did cooperate to complete the process. With regard to the time of assessment after the traumatic event, it has been shown that combat-related and home-coming effects persist on a range of psychosocial endpoints 20–30 years after exposure [41-43]. Also, longitudinal studies have shown that the psychological impact of war traumatic events on children persist for several years [40].

The strengths of the study include the fact that we assessed whole families, including all children in the home in face-to-face interviews, and correlated parent-child psychosocial outcomes. The assessment of all children in the home is rare in the literature, and it helped to offset the possible bias that could result from interviewing single children who may have special relations with their families [19]. In addition, our study involved a wide age range of offspring, who were assessed for several child outcomes, including anxiety, depression, deviant behaviour outside the home, adaptive behaviour, and adjustment within the family. In studying groups of children whose fathers had different levels of combat exposure, we were enabled to have adequate comparison groups, so that we could provide reliable data on the interaction of veterans' combat exposure and PTSD status with their children's psychosocial outcome.

### Father's combat exposure and PTSD status

With regard to our first hypothesis on the relationship between veterans' combat exposure/PTSD status and their children's psychosocial outcome variables, we found that combat exposure seemed to play a more significant role than PTSD. In this regard, it is noteworthy that there was no significant interaction between combat exposure and PTSD status for the 43 children whose fathers had both PTSD and POW status. The strength of combat exposure is shown by the fact that the children of the retired veterans consistently scored lowest on anxiety/depression and deviant behaviour, while having more positive scores on the subscales of adaptation and family adjustment (Table 4). However, these findings should be judged from the perspective that they seemed to have been influenced by the age of the father, the child's age and child's level of education. An implication of this ANCOVA finding is that, for this group of children, the experience and maturity that age tends to confer, coupled with better child formal education, could help to offset the possible adverse impact of their fathers' condition on their psychological functioning. There are conflicting reports in the literature on the issue of the impact of veterans' combat exposure and PTSD status on their children's psychological functioning. While some studies reported on the primacy of veterans' PTSD status [5,6], others found that veterans' combat exposure was positively correlated with hostility and violent behaviour among their children [9].

### Mother's characteristics: interaction with father's PTSD status

Our results were in support of the second hypothesis concerning the impact of the mothers' characteristics on children's outcome variables. We found that the mothers' PTSD status, anxiety, depression, and family adjustment were significantly correlated with the children's psychopathological status, behaviour, adaptation and family adjustment (Tables 5 &6). The mother's PTSD had a greater impact on the child outcome variables than the father's PTSD. Indeed, the group with father PTSD/mother no PTSD had significantly higher planful behavior than the group with father no PTSD/mother PTSD (P < 0.02), thus supporting a protective effect for mother's mental stability (Table 6). Again, the results of the ANCOVA analysis showed that it is possible that, with greater the experience and maturity that age tends to confer on the parents and the child, as well as better formal education for the child, it can be hoped that the child could overcome adverse family influences consequent on the parents' condition [44]. The results of the multiple regression analyses strengthened our observation of the primacy of the impact of the mother's characteristics (Table 7).

There is much support in the traumatology literature for our finding that the mothers' condition (especially anxiety) has a wide ranging impact on their children's psychosocial outcome [15,44-46]. This may have evolutionary [47] and biological [21] bases. In a study of offspring of holocaust survivors, it was found that maternal PTSD was particularly associated with their (non-PTSD) children having lower mean cortisol levels [21].

According to other reports, the factors that seemed to magnify the impact of veterans' condition on their children are veterans' abuse of alcohol and abusive violence on their wives [4,20]. The fact that these two factors were not much in evidence for the veterans in our study [27], probably contributed to the finding that the fathers' condition had less important association with the children's outcome variables. We conclude from this finding that, culture, per se, is not necessarily a protective factor; rather, it is the particular behaviour of significant adults in the child's life that impacts on the child's emotional functioning, behaviour and family adjustment. Although Arab scholars have advanced theories to show that the norms and dynamics of the culture are in support of our finding of the primacy of the mother's condition [48], we are more impressed by the concordance of our findings with biological studies [21,47].

### Abnormal test scores and co-morbidity

With regard to the prevalence of abnormal test scores, our finding about the commonness of anxiety-depression co-morbidity is in line with the literature [49,50]. We found the following frequencies: about 14% for anxiety/depression, 17% for deviant behaviour, 16.6%–19.7% for poor adaptive behaviour, and 9.6% – 23.1% for poor family adjustment. Some comparable general population data in the Arab world are available from the neighbouring United Arab Emirates. In a study of 2100 subjects aged 5.4 – 16.6 years, using Rutter B2 Scale, it was found that 13.5% showed some form of behaviour disorder [51]. From the same community in the UAE, using Rutter Parent Questionnaire, the rates of behavioural disorders reported were 11.8% to 16.5% [52,53]. The rates of DSM-IV disorders among children in the general population in the Al-Ain community ranged from 10.4% to 22.4% [52,54]. By comparison, in a study of 4500 youth aged 9–17 years in a rural community in the USA, it was found that 21.1% had at least one DSM-IV disorder (including: any depressive disorder, 2.9%; any anxiety disorder, 6.4%; and conduct disorder, 5.4%) [55]. Thus, our findings support the universality of childhood psychological experience for those in vulnerable family situations.

## Conclusion

Our findings support the impression that child emotional and behavioral experiences in vulnerable family situations transcend culture and are associated with the particular behaviour of significant adults in the child's life. The primacy of the mother's condition implies that interventions for children with these problems should include attempts to improve the psychological functioning of their mothers. Coupled with the finding of the positive influence of parental age, child's age and child's education, mental health education for these families has the potential to help those with psychosocial problems.

## Competing interests

The authors declare that they have no competing interests.

## Authors' contributions

FAA conceived and planned the study and supervised data collection, FAA and JUO did literature search, analyzed the data and wrote the manuscript. All authors read and approved the manuscript.
